# Assessing the value of objective suspicious transactions in Dutch anti-money laundering investigations: a network approach

**DOI:** 10.1140/epjds/s13688-026-00676-9

**Published:** 2026-06-25

**Authors:** Elena Candellone, Peter Gerbrands, Mahdi Shafiee Kamalabad, Javier Garcia-Bernardo

**Affiliations:** 1 Department of Methodology and Statistics, Utrecht University, Utrecht, The Netherlands; 2 Fiscal Information & Investigation Service (FIOD), Utrecht, The Netherlands; 3 Centre for Complex Systems Studies, Utrecht University, Utrecht, The Netherlands; 4 School of Economics, Utrecht University, Utrecht, The Netherlands; 5 ODISSEI SoDa Team, Utrecht University, Utrecht, The Netherlands

**Keywords:** Suspicious transaction reports, Money laundering, Network analysis, Financial intelligence, Anti-money laundering investigations

## Abstract

The increasing volume of suspicious transaction reports (STRs), covering transactions that may be generated by criminal activity, presents both opportunities and challenges for anti-money laundering (AML) investigations. Objective STRs, automatically triggered based on predefined criteria, are generally de-prioritized by investigators compared to subjective STRs filed on expert judgment. In this study, we analyze the investigative value of objective STRs using five years of data from the Dutch Anti-Money Laundering Centre, part of the Dutch Investigation Service for Financial and Tax Crime (FIOD), one of the national agencies involved in identifying and investigating money laundering. We apply a network science framework to analyze how objective STRs contribute to (i) expanding the number of unique entities linked to investigated cases, (ii) connecting previously disconnected dossiers, and (iii) improving predictive accuracy for identifying entities under investigation. Although objective STRs represent a small fraction of reports, they increase network coverage and connectivity, adding new entities and dossiers, including cases under investigation. However, including objective transaction data when computing centrality-based predictors does not improve the accuracy of identifying entities under active investigation. These findings suggest that, while objective reports help flag potential cases, more descriptive reporting is needed to support investigations.

## Introduction

Illicit activities generate massive financial flows, estimated to constitute 2% to 5% of global GDP, approximately $800 billion to $2 trillion [1]. Criminals must reintroduce these proceeds into the legitimate economy to be used in the financial system. This practice, although it is hard to legally capture [2], is known as *money laundering*. It is broadly defined as a process that aims to disguise the illegal origin of criminal proceeds [3], as codified in the Dutch Penal Code.^1^ Over the past decades, substantial collective efforts have been made to combat money laundering, such as the creation of the Financial Action Task Force (FATF) in 1989 [4]. Already in their first version of the 40 recommendations in 1990, the FATF recommended that financial institutions should report unusual transactions,^2^ which provides the primary source for our analysis. It has shaped global standards and focused on preventing drug traffickers and cartels from laundering money, counter-terrorist financing, and the proliferation financing of weapons of mass destruction [5].

In national contexts such as the Netherlands, these international recommendations and the associated European anti-money laundering regulations [e.g. 6] are translated into extensive legal and supervisory frameworks. The Money Laundering and Terrorist Financing (Prevention) Act (Wwft) [7] requires a range of reporting entities, such as financial institutions, crypto service providers, accountants, notaries, tax advisors, and real estate agents, among others, to monitor transactions and report unusual activity to the Financial Intelligence Unit (FIU-Nederland).^3^ Non-compliance has resulted in substantial fines for banks and service providers (e.g., ING was fined 775 million euros in 2018, ABN AMRO 480 million euros in 2021, Volksbank 20 million euros in 2025, and Rabobank was summoned by the Public Prosecution Service in 2025 [8–11]).

These measures have driven a rapid growth in reporting. Between 2021 and 2023, unusual transaction reports (UTRs) increased by 90% (from 1.23 million to 2.33 million). After an initial analysis, the Dutch Financial Intelligence Unit (FIU) reclassifies a subset as being suspicious. These suspicious transaction reports (STRs) nearly doubled (from 96,676 to 180,578) [5]. Such growth is often interpreted as stronger surveillance and more robust compliance systems, but it also creates a *data overload problem*: the exponential increase in reporting does not necessarily translate into proportional investigative value; the significance of the data is unknown a priori [12]. Instead, the FIU must screen large amounts of heterogeneous and frequently low-informative data before passing relevant cases to law enforcement institutions, including the Investigation Service for Financial and Tax Crime (FIOD).^4^ This, in turn, increases the analytical burden on investigators, who must prioritize cases under increasing complexity and volume. Moreover, as the FIU-Netherlands Annual Report of 2022 notes [13], reporting institutions differ in their internal guidelines, making reports difficult to compare systematically and weakening the coherence of the reporting ecosystem.

To face this issue, the Wwft distinguishes *objective* and *subjective* indicators for reporting unusual transactions. *Objective* reports are triggered automatically based on predefined criteria (e.g., cash transactions exceeding a threshold), while *subjective* reports are filed at the discretion of financial institutions. FIU-Netherlands suggests that objective reports often lack sufficient descriptive basis for opening investigations, whereas subjective reports are more likely to do so [13]. From a criminological perspective, this distinction echoes broader debates about automation versus discretion in surveillance and risk-based regulation [e.g. 14]: how much should detection rely on rules versus expertise? This debate has become increasingly relevant as reporting volumes rise and regulatory authorities explore the integration of automated detection tools with human oversight. In addition, both the objective and subjective reporting systems have their limitations and benefits [15]. The authors claim that although the objective system has a deterrent effect, forces money-launderers to choose more expensive and risky actions, and creates a clear paper trail, it leads to over-reporting and compliance-based responsibility-avoiding behavior. The subjective system fails to complete the paper trail and could lead to under-reporting due to cost-cutting or protecting confidentiality, yet it is more resource efficient and may provide details on how money-laundering takes place.

Given that in the Netherlands both types of reporting are used, this work focuses on the added value of objective reporting for investigation purposes and addresses the research question: *What is the investigative value of objective suspicious transaction reports for combating money laundering?*

**Figure 1:**
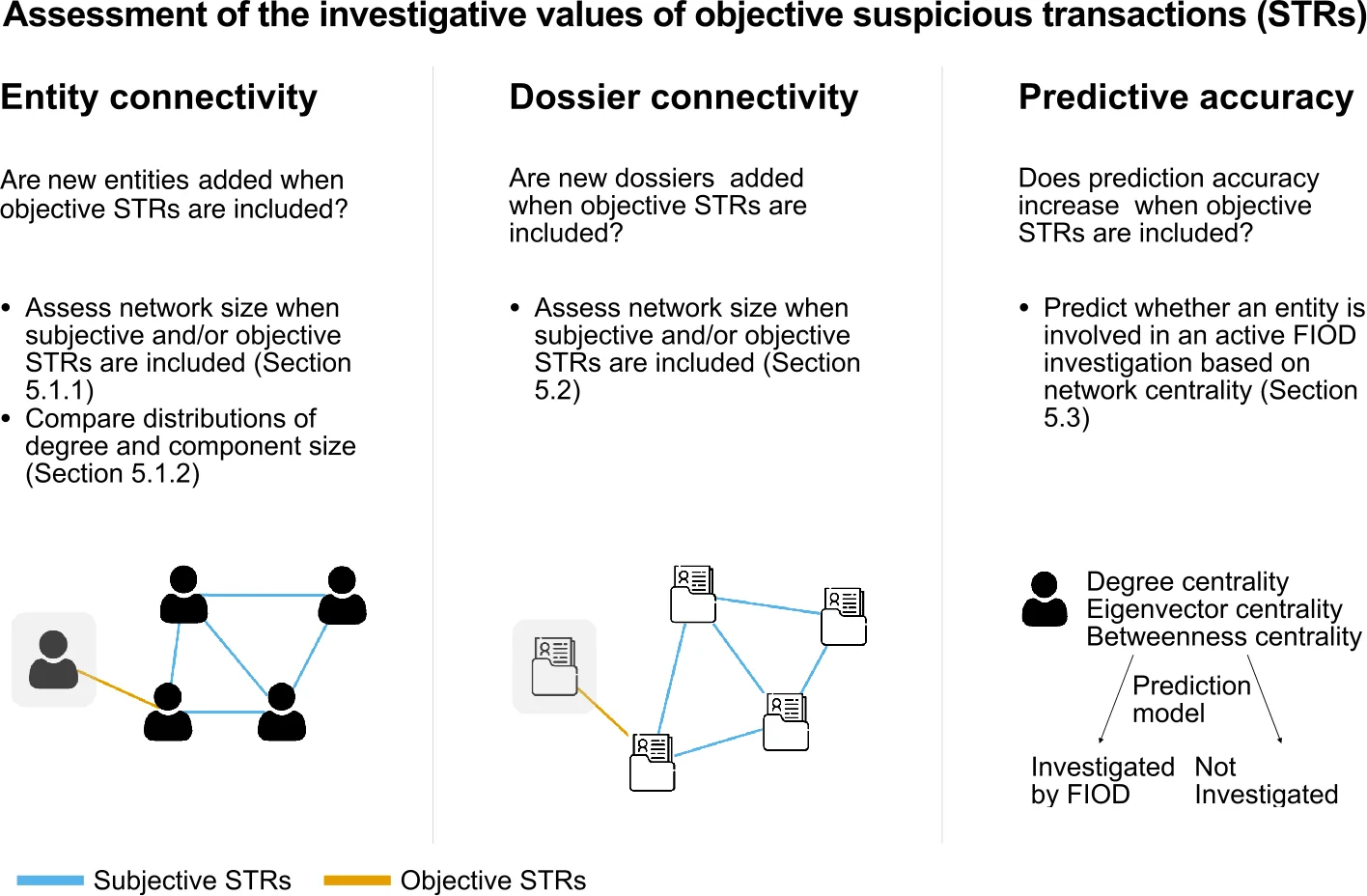
Scheme of investigative value assessment. We assess the added investigative value of objective suspicious transactions in three steps: *entity connectivity, dossier connectivity, and predictive accuracy*

We consider STRs to be of “investigative value” when they make a police investigation more effective and/or more efficient. In this case, effectiveness relates to an increased ability to identify (potential) money-launderers: the use of social network analysis is clearly demonstrated in the work of Duijn and Klerks [16], while efficiency focuses on the resources needed to identify them. As better explained in the next section, all actors need to be analyzed [17] to effectively analyze a criminal network. Therefore, we define the “investigative value” of objective STRs as the measurable contribution they make to expanding the connected structure of suspicious transaction networks, quantified through the increase of: *(i) entity connectivity*, i.e., the number of additional unique entities linked to investigated cases, and *(ii) dossier connectivity*, i.e., the number of previously disconnected dossiers joined into the main investigative network. The efficiency of a criminal analysis is related to the resources required for the investigation. Thus “investigative value” is also improved when the *(iii) predictive accuracy* in identifying the role and importance of entities under investigation based on their centrality in the suspicious transaction network is improved (see Fig. 1 for a schematic representation of how we operationalize investigative value).

To answer our research question, we adopt a network science approach. Examining money laundering through the lens of network science enables us to shift from localized investigative methods to a complexity-driven perspective that captures the systemic nature of financial crime. This shift towards a network science approach has been extremely useful as criminal groups do not act in isolation but operate within structured, often resilient networks that facilitate the movement, concealment, and redistribution of illicit profits.

We apply these methods to a unique dataset of suspicious transactions reported in the Netherlands. These data have been provided through a collaboration with the Dutch FIOD (the Investigation Service for Financial and Tax Crime), and they contain information on the report type (i.e., objective or subjective) of each transaction. The dataset is explained in detail in Sect. 3. To the best of our knowledge, no datasets used in prior research contain STR-level information with a distinction between report types. Our study, therefore, contributes the first comparative assessment of subjective versus objective indicators of suspicious transactions within a network science perspective that integrates entity-level, dossier-level, and predictive analyses. We provide an evidence-based evaluation of how objective reports complement subjective ones in supporting anti-money laundering investigations.

Our assessment strategy is organized into three components. First, we evaluate the *(i) entity connectivity* of objective STRs by counting how many entities are involved in only objective STRs (Sect. 5.1.1) and by comparing the network degree and component size distributions (Sect. 5.1.2). Second, we assess the *(ii) dossier connectivity*, by identifying the dossiers composed of only entities involved in objective STRs and comparing the network component sizes (Sect. 5.2). Third, we determine the *(iii) predictive accuracy* of network centrality measures by fitting a logistic regression model and Random Forest to predict whether an entity is involved in an active FIOD investigation (Sect. 5.3).

The rest of the paper is organized as follows. Section 2 describes the literature. Section 3 describes the data and the processing steps used to construct entity and dossier networks. Section 4 outlines our methodological approach. Section 5 presents the results of our analyses. Finally, we discuss the investigative relevance of our findings in Sect. 6.

## Related work

The criminological literature has long recognized that criminal groups do not operate in isolation. Already in 1988, Barry Leighton published on the community concept in criminology [18]. As a result, network-based approaches have become central to understanding how criminal systems function and how they may be disrupted. Prior criminological research has analyzed crime from a network perspective [19–33]. This perspective has been applied to Mafia groups [19, 22, 23, 28, 29], drug cartels [30, 34], and terrorist organizations [20]. Most of these studies treat the crime network as a social network, where individuals are connected through interactions, such as family relationships, hierarchical organizational structures, sharing monetary transactions, or alliances/fights. Yet, analyzing the hierarchy of a criminal organization is not sufficient anymore since criminal organizations are developing into a decentralized network form [17], which emphasizes the need to analyze all actors in the network.

In parallel, research on dark-web networks and online illicit markets extends network-based approaches to digital ecosystems. Studies on dark web marketplaces networks [24, 32] demonstrate how connectivity shapes both criminal opportunities and vulnerabilities. As mentioned earlier [17], this work also consistently shows that illicit networks often combine centralized coordination with decentralized operational layers, resulting in systems that are simultaneously efficient and resilient.

Within the domain of money laundering, network analysis has emerged as a promising tool for understanding transaction-based patterns, identifying suspicious behavior, and assessing systemic risks. Prior work has investigated transaction networks [35], measured the centrality of agents involved in illicit flows [36–39], evaluated the robustness of money laundering structures [25, 31], and examined the effects of regulatory changes on network topology [3]. Other studies use graph-based machine learning, applying link prediction or anomaly detection to uncover hidden relationships or suspicious transactions [40–42]. These computational approaches have advanced detection strategies, but their performance depends heavily on the availability and granularity of high-quality transaction data.

A persistent challenge is the limited accessibility of detailed financial datasets. As Deprez et al. [35] note, many studies rely on proprietary data from individual banks and therefore analyze only unusual transaction reports (UTRs), or from police registers [43]. A smaller number of studies use synthetic datasets [44] or leaked documents, such as the Panama Papers [45], each with important limitations regarding realism or representativeness. Crucially, existing research often lacks suspicious transaction reports (STRs) and therefore cannot distinguish between different reporting mechanisms. Although a substantial body of literature exists on crime networks, empirical comparisons between different types of indicators of suspicious transactions remain limited, and little work evaluates how various report types contribute to investigative processes when embedded in network structures.

Our work addresses this gap by providing the first network-science-based assessment of the investigative value of objective STRs. By integrating entity-level network measures, dossier connectivity, and predictive modeling, we position the distinction between objective and subjective reporting within a structural analysis of money-laundering networks and provide an empirically grounded evaluation of their respective contributions to financial crime investigation.

## Suspicious transactions reports: data origin and processing

The Money Laundering and Terrorist Financing (Prevention) Act (Wwft) [7], which was issued as part of the Dutch legislation in 2008, provides a framework for institutions, such as banks and tax advisors,^5^ to report unusual transactions. Such a reporting procedure is a fundamental step in a collaborative effort between institutions and the government to fight money laundering and other forms of financial crime. To clarify the origin and intention of such reports, the Dutch Financial Intelligence Unit (FIU) divided unusual transactions to be reported by institutions into two main categories: *objective* and *subjective* transactions. The *objective* unusual transactions are based on objective indicators provided in the Wwft (e.g. “A transaction for an amount of €10,000 or more, involving cash exchange into another currency or from small to large denominations”). In contrast, the *subjective* transactions “give reason to assume that the transaction may be related to money laundering or the financing of terrorism” [7]. The FIU classifies unusual transactions as suspicious or not suspicious, based on internal criteria, and reports suspicious transaction reports (STRs) to law enforcement. The STRs include a wide range of details about the actors involved in suspicious transactions, such as addresses, bank account numbers, company ownership, transaction descriptions, amounts, and timestamps.

In this study, we analyze a dataset of STRs that have been shared with Dutch law enforcement in the period 2018–2023, encompassing 333,757 transactions from 1991 to 2023. The dataset is subdivided into 331,747 reports and 52,672 dossiers (i.e., every dossier contains one or more reports, each containing transactions). These transactions involve 166,377 entities, 76.4% of which are individuals (127,138) and 23.6% companies (39,239). We exclude companies and individuals related to the Dutch Tax Authority (*De Belastingdienst*) on the following basis: all companies with “*Belastingdienst*” in their name; all companies with the legal form “*Naamloze vennootschap*” (i.e., publicly traded companies such as major banks and insurers); all companies that are legal entities under public law (e.g., local or national government agencies, municipalities, ministries). Moreover, we identified and excluded any bank accounts linked to these companies, and further excluded any individuals associated with those bank accounts. This results in excluding 155 individuals and 369 companies, respectively 0.1% and 0.9% of all individuals and companies. These entities appear because other actors make payments to them, but they do not provide investigative value, so we exclude them. After removing these entities, we are left with 165,853 entities and 333,025 transactions. Importantly, the data include information on individuals and companies involved in active investigations by FIOD, accounting for 541 companies and 1895 individuals (1.4% of all companies and 1.5% of all individuals). We use it as a proxy to understand the value of objective versus subjective transactions for law enforcement investigations, using the label “investigated/not investigated” as the outcome variable of the prediction model (see Sect. 5.3).

**Figure 2:**
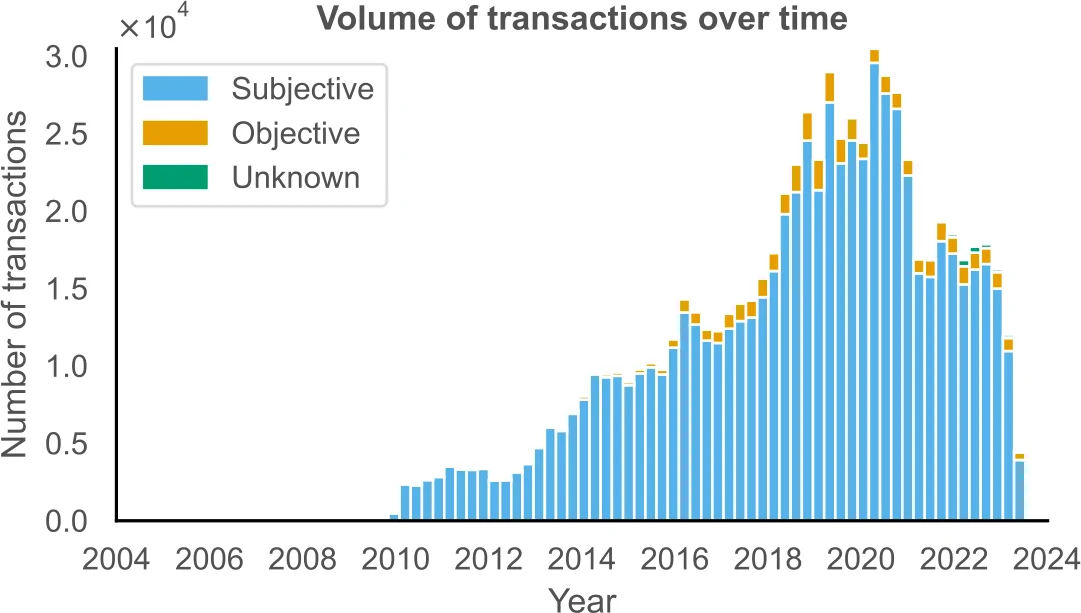
Subjective transactions show the steepest growth over time. Number of transactions over time in the dataset, divided per transaction type: subjective (blue), objective (orange), unknown (green). Note that even though we have access to the reports of the last five years (2018–2023), transaction dates are not limited to that period. The x-axis is limited to those years with more than one transaction

The number of transactions is largest for the reporting period (2018–2023), although the transactions themselves may have occurred earlier (Fig. 2). Regarding the types of transactions, the dataset comprises 310,437 (93.2%) subjective, 21,398 (6.4%) objective, and 1190 (0.4%) unknown suspicious transactions. Subjective suspicious transactions experienced the steepest growth over time (Fig. 2), which is related to both the increase in UTRs and the classification of transactions as suspicious or not-suspicious by the FIU. Of all the dossiers, around 80% contains only subjective transactions, while a smaller share includes only objective (around 13%) or unknown transactions. A small portion of the dossiers combines different types of transactions. Table 1 summarizes these distributions, along with the corresponding number of transactions they account for.

**Table 1:** Summary statistics of dossiers and transaction types

Dossier type	Number of dossiers	Number of transactions
Only subjective transactions	42,024 (79.9%)	262,889 (84.7% of all subjective)
Only objective transactions	6816 (13.0%)	10,112 (47.3% of all objective)
Only unknown	74 (0.1%)	716 (60.2% of all unknown)
Mixed dossiers	3689 (7.0%)	47,394 subj. (15.3%) and 11,286 obj. (52.7%)
Total	52,614	333,025

We exclude transactions of unknown type from all network constructions, as these refer to transactions reported within the reporting system of the Dutch Caribbean islands and are not relevant to the objective/subjective distinction that is the focus of this study. Consequently, the 74 dossiers composed exclusively of unknown transactions do not appear in any network.

Mixed dossiers are fully retained in the analysis: all objective transactions they contain contribute to the Objective and Complete networks, and all subjective transactions contribute to the Subjective and Complete networks. Beyond this transaction-level classification, we also treat transactions as interactions among entities, motivating the construction of an entity transaction network described in the next section.

## Methods

### Constructing the entity network from suspicious transaction reports

**Figure 3:**
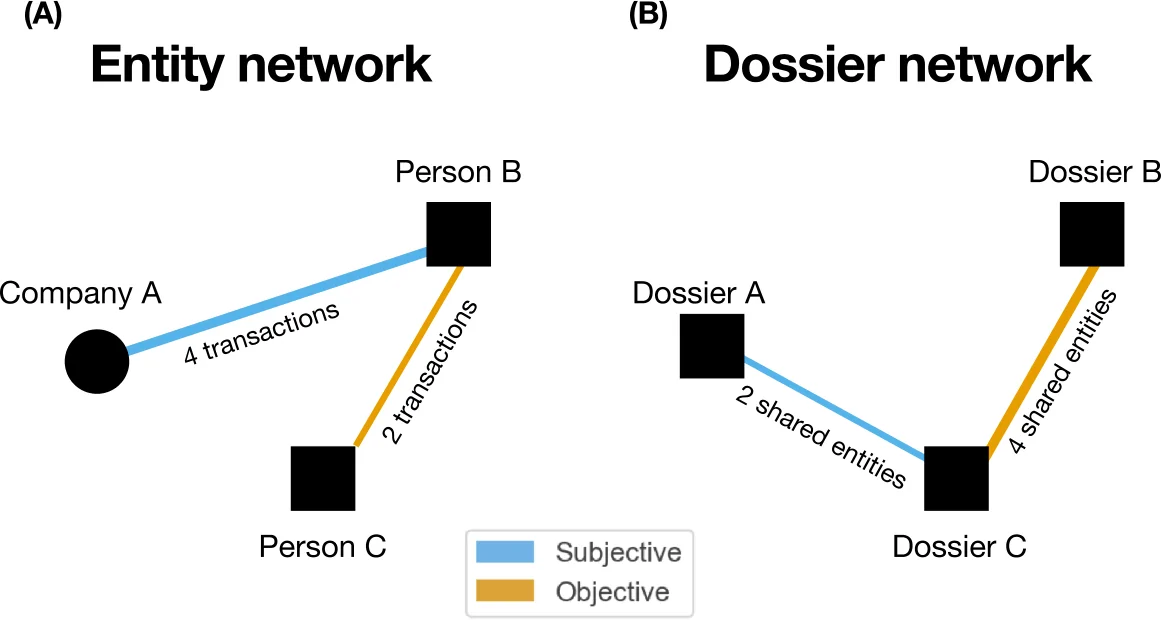
Schematic representation of the entity and dossier network. Panel **(A)** shows the entity network, where nodes are companies or people, while edges are transactions, including the sender and receiver as nodes connected by the edge. If a transaction involves multiple senders and/or receivers, we create one edge for each combination of sender and receiver. The edge weight is given by the number of transactions between the two entities. The edge colors represent the transaction type: subjective (blue) and objective (orange). Panel **(B)** shows the dossier network, where the nodes are dossiers while the edges are shared entities (i.e., two entities that appear in both dossiers)

We construct entity networks where nodes represent entities (companies or individuals), and an edge is present between two entities if they are involved in a transaction either as a sender or receiver. The edge weight is given by the number of transactions between two entities. We treat the edges as undirected, disregarding the sender and receiver distinctions, as aggregated transactions reflect the overall relationships between entities. This choice does not affect the study’s results. Figure 3A shows a schematic representation of the entity network.

We measure two key structural network properties, to quantify the relevance of subjective versus objective transactions: *weighted degree*, i.e., the number of connections weighted for the number of transactions per connection each node has, indicating an entity’s level of activity in transaction, and *connected component size*, i.e., the number of entities reachable from a given starting point, reflecting the potential basin of reference of an investigation. To further characterize network connectivity, i.e., to assess the investigative value globally, we fit candidate distributions to the degree sequences of the networks. We use a maximum-likelihood-based method that selects the most appropriate distribution while maximizing the portion of the data included in the fit [46]. For each network, we estimate the scaling exponent *α* and lower bound $x_{min}$, with the power-law probability density function defined as

1 $$ p(x)={\frac {\alpha -1}{x_{\min }}}\left ({\frac {x}{x_{\min }}} \right )^{-\alpha}. $$

The results are discussed in Sect. 5.1.2. In the next section, we describe the dossier networks.

### Dossier network

In Sect. 3, we describe the data structure, mentioning that suspicious transactions are reported in batches known as Suspicious Transaction Reports (STRs). These reports are collected in dossiers, a more coarse-grained subdivision that encapsulates some investigation rationale, namely, the assumption that transactions found in the same dossier might be related to the same entities and/or the wider money flow scheme. We then investigate the impact of adding information about objective transactions at this more coarse-grained level, i.e., analyzing the difference between relationships among dossiers when adding objective transactions.

We construct a dossier network to study this phenomenon: nodes represent dossiers, and an edge is created between two dossiers that share entities (person or company). Dossiers linked via shared entities may indicate overlapping or related criminal schemes. The number of shared entities accounts for the edge weight. The network creation process follows these steps: we first map each transaction (and related entities) to its corresponding dossier. Then, we create an incidence matrix, where rows are dossiers and columns are entities, and the presence or absence of an entity in a dossier determines each entry. The incidence matrix is projected to a dossier-dossier adjacency matrix to generate the dossier network. Figure 3B shows a schematic representation of the dossier network. Results are discussed in Sect. 5.2. In the next section, we define network centrality measures and the prediction models.

### Centrality measures and prediction metrics

Moving to a more fine-grained level, we are interested in comparing the relevance of nodes across different network structures, and therefore, we study several network centrality measures. Network centrality allows us to compare, for example, which entities act as intermediaries of suspicious transactions. Here, we define the measures used:

- *degree* centrality measures how connected a node is to the network. It corresponds to the degree of a node, i.e., the number of edges incident on that node, weighted by the edge weights. In transaction networks, degree centrality can be interpreted as the volume capacity of a certain node, i.e., the number of transactions in which the entity is involved.
- *betweenness* centrality quantifies the extent to which a node acts as a bridge between other nodes in the network. Betweenness centrality is defined as the number of shortest paths $\sigma _{st} \left ( v \right )$ from node *s* to node *t* passing through *v*, divided by the total number of shortest paths between those nodes $\sigma _{st}$, i.e.,
  2 $$ g_{d} \left ( v \right ) = \sum _{s \neq t \neq v} \frac{\sigma _{st} \left ( v \right )}{\sigma _{st}}. $$
  In transaction networks, betweenness centrality can be interpreted as identifying the nodes that facilitate the money flow, e.g., financial institutions or professional money laundering facilitators.
- *eigenvector* centrality assesses a node’s influence by considering both its direct connections and the importance of those. Nodes linked to highly central entities receive higher scores. Mathematically, the eigenvector centrality **x** satisfies $A \mathbf{x} = \lambda \mathbf{x}$, where *λ* is the largest eigenvalue of *A* and **x** is the corresponding eigenvector. In transaction networks, eigenvector centrality can be interpreted as a measure of the influence of nodes, i.e., those entities that are involved in transactions with other influential nodes.

We then evaluate the ranking quality using a tool from information retrieval: the normalized discounted cumulative gain (NDCG) [47]. It is a score that quantifies how early relevant documents appear in search engines. In our context, it accounts for the effectiveness of each centrality measure’s ranking in finding entities involved in investigations with high centrality positions. The NDCG is defined as follows. Given a ranking of entities, each assigned a relevance score $r_{i} $, i.e., the importance of the entity to the investigation (e.g., binary relevance where $r_{i} = 1 $ if the entity is involved in the investigation, and $r_{i} = 0 $ otherwise), the discounted cumulative gain (DCG) at position *k* is calculated as

$$ \mathrm{DCG}_{k} = r_{1} + \sum _{i=2}^{k} \frac{r_{i}}{\log _{2}(i+1)}, $$

which penalizes lower-ranked positions with a logarithmic penalty. The ideal DCG (IDCG) is computed by ordering entities in the optimal ranking by decreasing relevance. NDCG is then obtained by normalizing the DCG by the IDCG:

$$ \mathrm{NDCG}_{k} = \frac{\mathrm{DCG}_{k}}{\mathrm{IDCG}_{k}}, $$

resulting in a score between 0 and 1 that quantifies how well the ranking prioritizes relevant entities early in the list.

Finally, we evaluate how well network structure and centrality measures can predict investigative involvement by fitting a logistic regression model and a Random Forest classifier [48]. The outcome variable is binary, i.e., whether an entity is under active investigation by FIOD (y=1) or not (y=0). Before fitting, we assess multicollinearity among predictors using the Spearman correlation [49]. The predictors are degree, betweenness, and eigenvector centrality, standardised before fitting.

Model performance is assessed using the Area Under the Receiver Operating Characteristic Curve (ROC AUC) [50], which quantifies the model’s ability to discriminate between investigated and non-investigated entities across all possible classification thresholds. It can be interpreted as the probability that a randomly chosen investigated entity is ranked higher than a non-investigated one. An $\text{ROC AUC} = 0.5$ is equal to a random prediction, whereas $\text{ROC AUC} = 1$ means perfect discrimination.

To obtain unbiased AUC estimates, we adopt a nested cross-validation scheme with a 5-fold outer loop for evaluation and a 3-fold inner loop for model selection. For logistic regression, the optimal feature subset is selected within each inner fold; for the Random Forest, hyperparameters (number of trees, maximum depth, minimum samples per split and per leaf, and number of features considered at each split) are tuned via exhaustive grid search within the same inner loop. Performance is reported as the AUC computed on the aggregated out-of-fold predictions of the outer loop. To formally compare AUC scores across network types and between models, we apply the DeLong test [51], which provides a non-parametric statistical test for the difference between two correlated ROC curves.

The results of the centrality measure ranking comparison and prediction models are presented and interpreted in Sect. 5.3.

## Results and discussion

Here, we present the results and discuss their implication with a three-step assessment of the investigative value of objective STRs. Section 5.1 presents the results of the *entity connectivity* step, where we assess whether adding objective STRs adds entities to the network of suspicious transactions; Sect. 5.2 shows the results of the *dossier connectivity* step, where we discuss whether adding objective STRs adds dossiers to the network of suspicious transactions; finally, Sect. 5.3 shows the results of the *predictive accuracy* step, where we compare the rankings from different centrality measures. We use them as predictors of involvement in FIOD investigations.

### Entity connectivity

#### Entities in subjective vs. objective transactions

Our work aims to answer the following research question: *What is the added investigative value of objective transaction reports for combating money laundering?*

**Figure 4:**
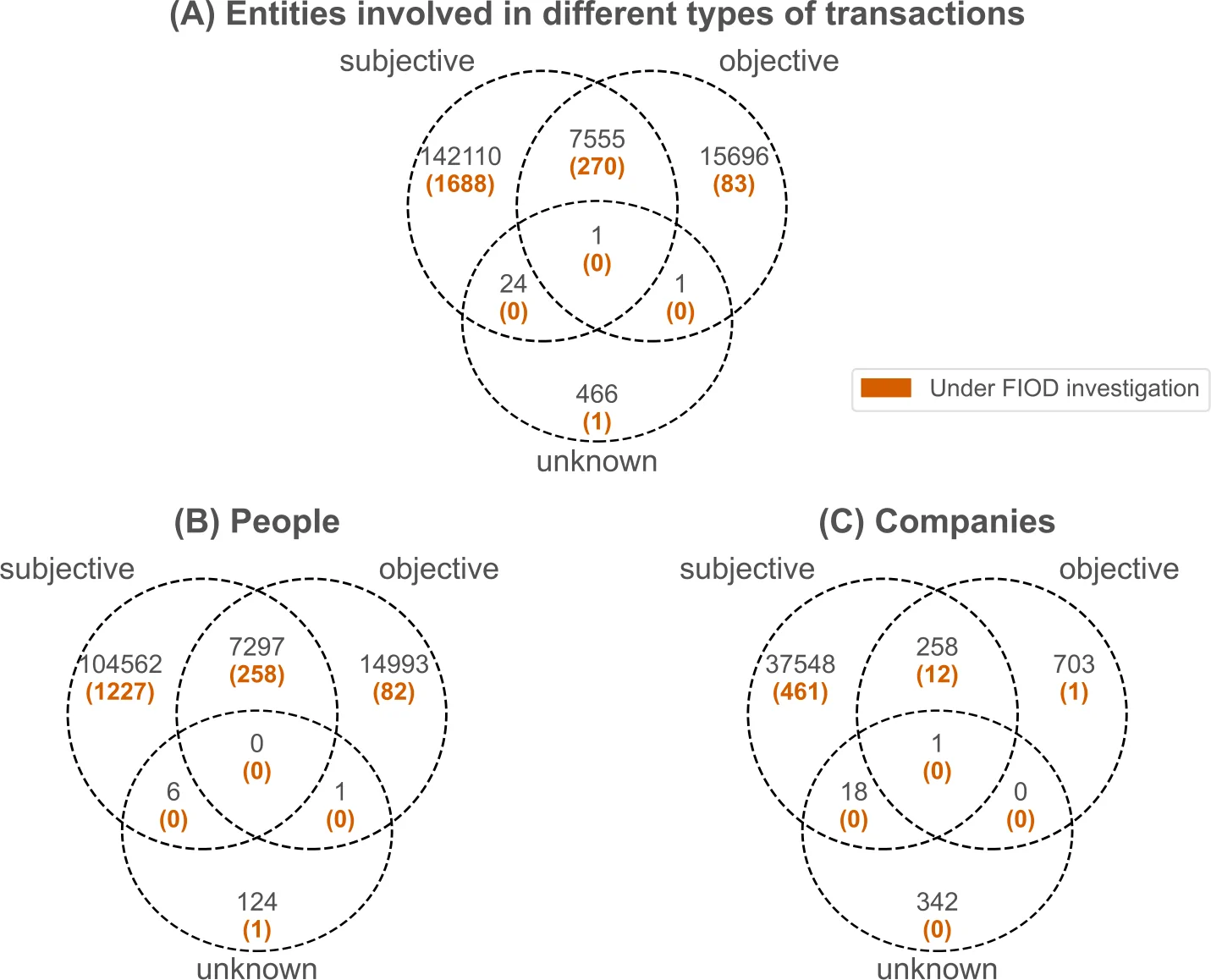
Objective transactions reveal new entities, especially individuals. Venn diagrams illustrating the number of entities involved in various types of transactions (subjective, objective, and unknown). Panel **(A)** shows all the entities together, while the bottom panels differentiate between entity types: Panel **(B)** for persons, and Panel **(C)** for companies. Numbers in parentheses represent how many of each category are involved in FIOD investigations

First, we count the number of entities involved in each of the two transaction types (Fig. 4). We find that 4.56% (7555) of all entities appear in both subjective and objective reports, representing 32.5% of the entities involved in objective transactions. Moreover, 9.46% (15,696) of all entities participate only in objective transactions, making up the remaining 67.5% of entities involved in objective transactions. Among this group, 83 entities are investigated by FIOD (i.e., 5% of those involved only in objective transactions). This suggests that focusing investigations only on subjective transactions overlooks a substantial share of entities involved only in objective transactions. Specifically, 3.4% of entities currently under investigation would be missed. Individuals are proportionally more involved in objective transactions than companies (Fig. 4B-C). 11.81% (14,993) of individuals engage only in objective transactions, compared to 1.81% (703) of companies. This discrepancy may stem from companies being more affected by regulatory thresholds that trigger classification as objective transactions. Moreover, many objective reports are related to cash transactions, which are more frequently attributed to individuals than to companies.

#### Comparing entity connectivity: degree and component size distributions

We further analyze connectivity patterns in entity networks resulting from suspicious transaction types to determine whether objective transactions offer a meaningful complement to subjective transactions. We show that incorporating objective transactions increases the number of entities connected, which could aid intelligence investigators in connecting otherwise unrelated entities.

We generated three types of entity networks as described in Sect. 4.1: the *complete* network, i.e., including both subjective and objective transactions, the *subjective* network, i.e., including only subjective transactions, and the *objective* network, including only objective transactions. Compared to using only subjective transactions, using the full network increases the number of nodes by 15,403 (83 of which are investigated by FIOD) and the number of edges by 17,171 (Table 2).

**Table 2:** Descriptive statistics for each network type. Note that the number of edges does not correspond to the number of transactions, as we count unique edges between pairs of entities

Network type	Number of nodes (entities)	Number of edges (transactions)
Complete	152,337 (1926 investigated)	184,363 (165,487 subj., 17,171 obj., and 1705 both)
Subjective	136,934 (1843 investigated)	167,192
Objective	22,311 (307 investigated)	18,876

**Figure 5:**
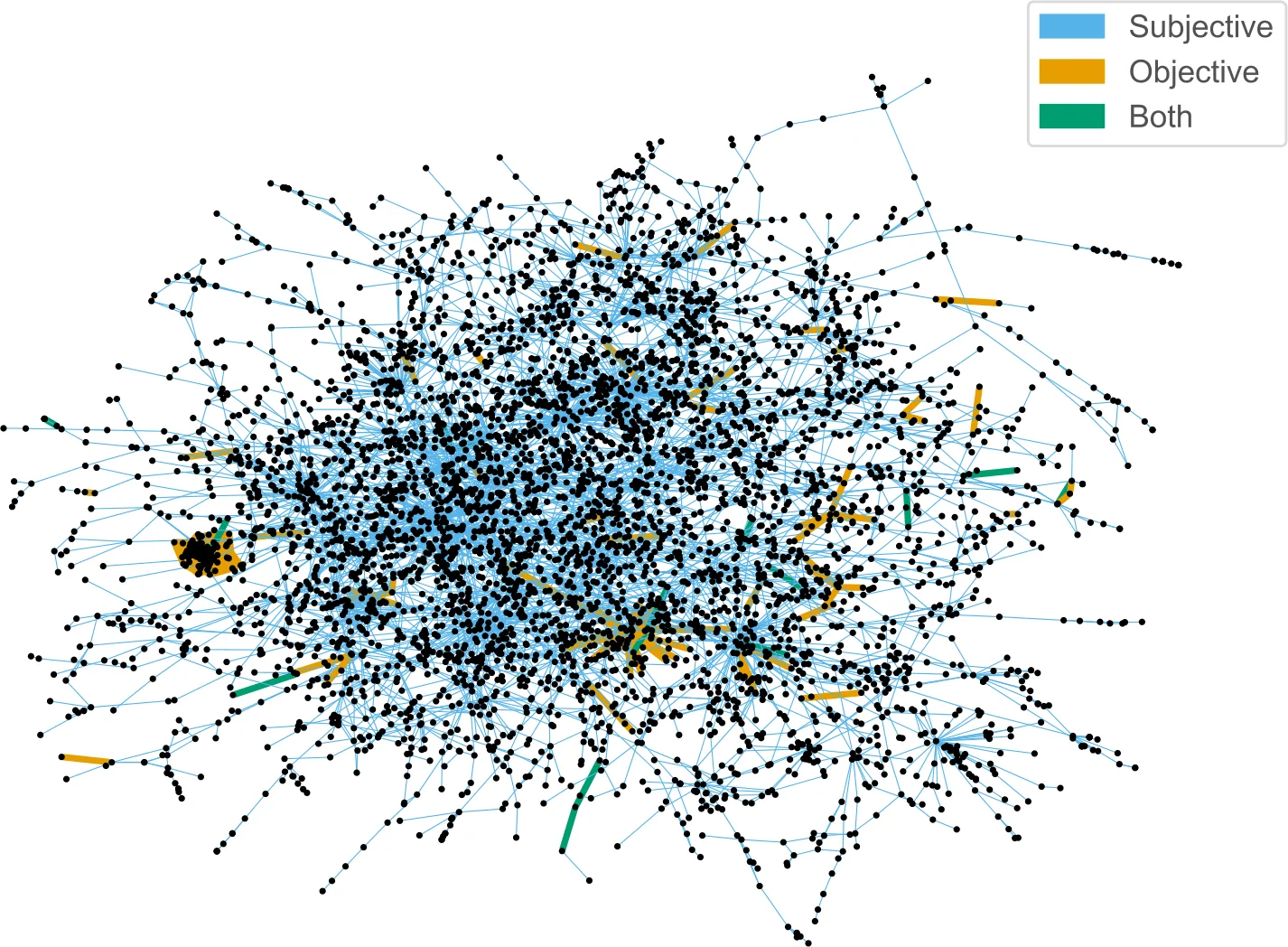
Entity network visualization. Using the Python package *igraph* [52], we visualize a random sample of the network, choosing randomly 15% per edge type and then limited to the giant component. The edge color is given by the transaction type (blue for subjective, orange for objective, and green for both transaction types), and the edge width of subjective edges is made smaller to make the other edge types more clearly visible

From a visual standpoint, the integration of objective transactions enhances the network’s connectivity (Fig. 5). Examining the giant component (i.e., the largest connected component in Fig. 5) reveals several qualitative insights: *(a)* there exists a cluster of densely connected nodes (hubs) characterized by numerous incoming and outgoing transactions; *(b)* a significant portion of nodes are situated peripherally, and *(c)* objective transactions primarily facilitate connections from peripheral nodes to the network’s main structure, while only a few objective connections are associated with hubs.

**Figure 6:**
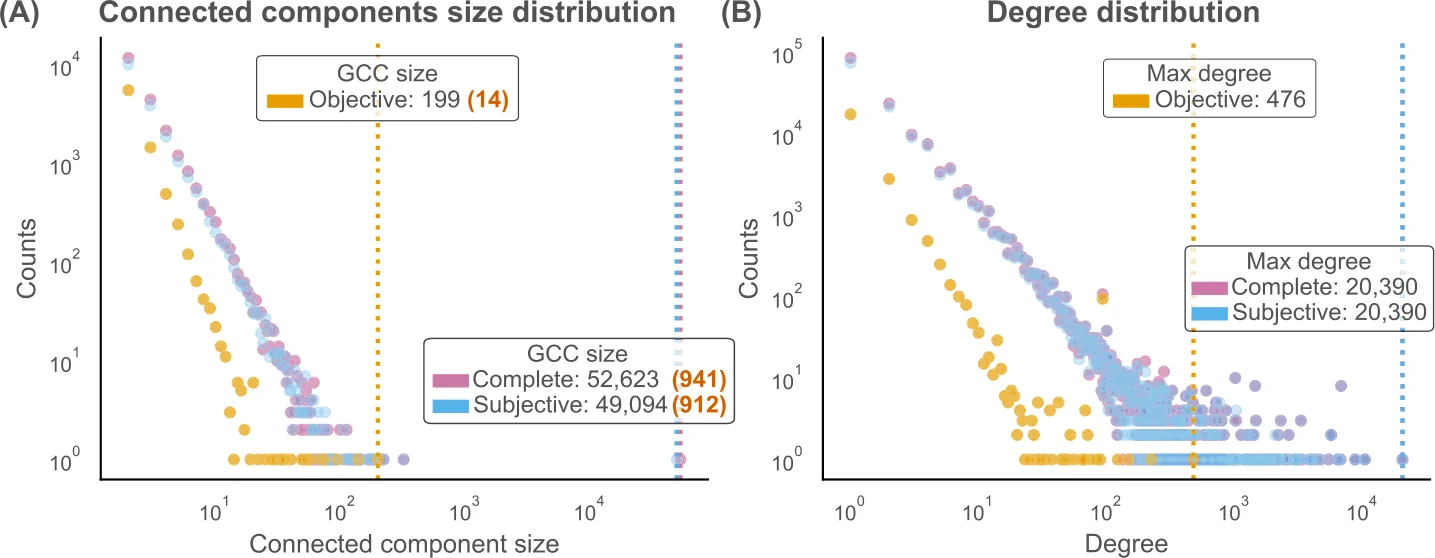
Distributions of network measures: connected component size and node degree. Panel **(A)** shows the connected component size distributions for the three networks (subjective in blue, objective in orange, and complete in purple, respectively). Panel **(B)** shows the degree distribution for the same networks. Dotted vertical lines represent the maximum value measures, respectively, the giant component size and the maximum degree. Numbers in parentheses represent how many of each category are involved in FIOD investigations

While these are qualitative observations based on Fig. 5, we further investigate network properties by looking at distributions of the connected component size (Fig. 6A) and node degree (Fig. 6B) for each network. The goal is to quantify the extent to which adding objective transactions increases connectivity, both globally (i.e., by increasing the number of nodes connected to a network component) and locally (i.e., by increasing the number of edges per node).

We find that in the subjective and complete graph, the giant component (i.e., the biggest connected component) contains respectively 35.9% and 34.5% of nodes, while in the case of the objective network, there is no such outstanding giant component, as the largest component contains only 199 nodes (Fig. 6A). The first two networks show a behavior common to socioeconomic networks, where most of the smaller components contain a few nodes, while the giant component comprises the majority of the connections. Looking at the degree distributions, we find that the best fit for the three cases is a power-law distribution with exponent 2<α<3, as shown in Table 3. This is a so-called heavy-tailed distribution, where many nodes have few connections and a few nodes have many (i.e., hubs), confirming the qualitative insights from visual inspection of Fig. 5.

**Table 3:** Exponent (*α*) of the degree distributions visualized in Fig. 6B, estimated using [46]

	$x_{\mathrm{min}}$	Distribution	*α*
Complete	1	Powerlaw	2.19
Subjective	3	Powerlaw	2.40
Objective	1	Powerlaw	3.00

Finally, while the objective network does not show strong connectivity patterns on its own (i.e., no outstanding giant component), incorporating the information of objective transactions helps connect an additional 3529 nodes to the giant component of the complete graph. This corresponds to 7.2% new nodes, of which FIOD investigated 29. We can thus conclude that objective transactions hold some investigative value from the *entity connectivity* perspective. In the next section, we further characterize and assess the *dossier connectivity*.

### Dossier connectivity

In this section, we create dossier networks to test whether adding objective transactions increases connectivity among investigation batches (i.e., the dossiers). The three dossier networks are generated similarly to the entity networks: the *complete* network, i.e., including both entities involved in subjective and objective transactions, the *subjective* network, i.e., including only entities in subjective transactions, and the *objective* network, including only entities in objective transactions. Table 4 shows the descriptive statistics of these networks. We compare the *complete* and *subjective* dossier networks, noticing that the inclusion of objective transactions provides links to open investigations. The addition of objective transactions adds 6816 new dossiers to the complete network (12.97% of the total number of dossiers), of which 235 contain FIOD-investigated entities, and 15,697 new entities (9.49% of the total number of entities), of which 83 are investigated. Moreover, there is an increase in the giant component size too, with 1479 new dossiers (9.67% of the total number of dossiers) added to the biggest connectivity cluster, among which 127 contain investigated entities. In summary, the inclusion of objective transactions increases the *dossier connectivity* and contributes to a larger giant component. In the next section, we assess the *predictive accuracy* difference between network types.

**Table 4:** Dossier network descriptive statistics for each network type

Network type	Number of nodes (dossiers)	Number of edges (shared entities)	Giant component size
Complete	52,538 (3935 with investigated ent.)	582,931	16,778 (2506 with investigated ent.)
Subjective	45,722 (3700 with investigated ent.)	547,315	15,299 (2379 with investigated ent.)
Objective	10,505 (629 with investigated ent.)	20,925	290 (35 with investigated ent.)

### Predicting investigated entities with network centrality

This section investigates the varying levels of importance (centrality) of nodes across different network types. Moreover, it examines the extent to which these centrality measures can predict an entity’s involvement in the context of FIOD investigations.

We compute the centrality measures explained in Sect. 4.3, i.e., *degree*, *betweenness*, and *eigenvector* centrality. We rank each entity based on each centrality measure within the different network types to determine their relative importance. We examine the top 10 nodes based on their ranks in the objective network to understand to what extent objective STRs can aid investigations.

Only three of the ten highest-degree nodes in the objective network also appear in the subjective network (Table 5). Their centrality increases in the transition from the subjective to the complete network, as shown in Table 5 (e.g., node 31 rises from rank $10{,}840^{th}$ to 274th place). However, in absolute terms, the top 10 nodes by degree centrality in the objective network are not central in the complete network. A similar pattern emerges for eigenvector centrality (Table 6): five of the top ten nodes also appear in the subjective network and experience a corresponding increase in ranking. However, none become highly central in the complete network. In contrast, nearly all top nodes by betweenness centrality (Table 7), except one, are present in both networks. Moreover, node 31 shows the most radical improvement: it is the most central in the objective network and becomes the $23^{rd}$-ranked node by betweenness in the complete network. This node is related to gambling activities that often involve cash transactions, explaining why adding objective STRs increases its centrality. Such companies typically report STRs themselves and are included as parties to the transaction. The entity of interest for further investigation is often the gambler (i.e., the transaction sender), not the company (i.e., the transaction receiver), which is likely not directly involved in criminal activity. This illustrates a broader interpretive challenge: objective STRs can enrich network connectivity and surface structural bridge nodes, but high centrality in this context reflects reporting patterns rather than criminal relevance.

**Table 5:** Top 10 nodes by *degree* centrality

Degree centrality			
entity id	complete	subjective	objective
31	274	10,840	1
34	651	-	2
13	962	99,781	3
6	1151	-	4
19	1528	-	51
16	1528	-	51
17	1528	-	51
18	1528	-	51
35	1301	10,009	51
20	1528	-	51

**Table 6:** Top 10 nodes by *eigenvector* centrality

Eigenvector centrality			
entity id	complete	subjective	objective
31	5307	14,085	1
6	9931	-	2
3	10,481	81,368	3
5	11,629	-	4
12	11,579	81,368	4
9	12,128	81,368	6
1	12,296	-	7
2	12,591	-	8
14	12,591	-	8
10	12,527	13,699	10

**Table 7:** Top 10 nodes by *betweenness* centrality

Betweenness centrality			
entity id	complete	subjective	objective
31	23	4182	1
32	1	1	2
30	1753	-	3
4	1618	16,859	4
33	408	492	5
11	978	640	6
0	14,595	14,876	7
13	4376	84,787	8
8	996	4180	9
7	324	18,166	10

The analysis shows that while a few nodes gain importance during the transition from the objective to the complete network, the most central nodes in the objective network typically do not hold central positions in the complete network. Still, in some limited cases, centrality within objective transactions does translate into increased importance in the complete network.

**Figure 7:**
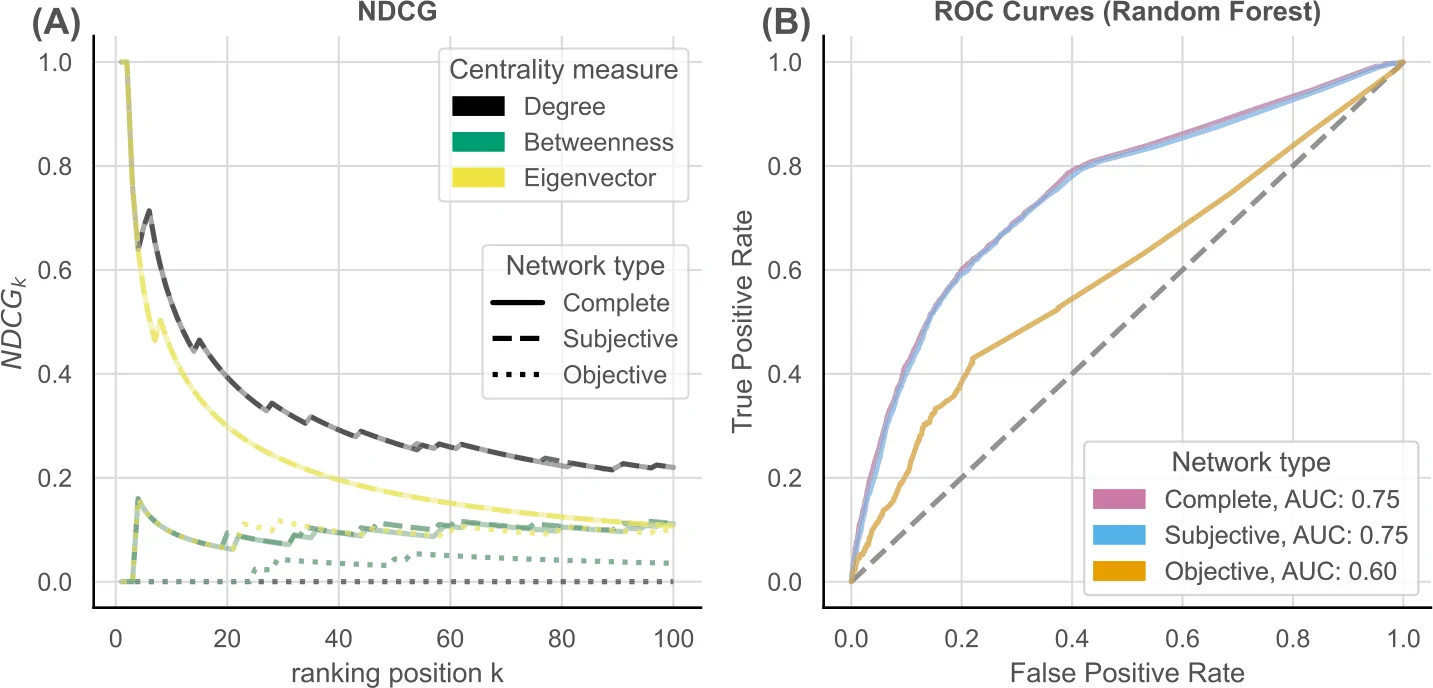
Node centrality assessment using information retrieval and Random Forest. Panel **(A)** shows the normalized discounted cumulative gain (NDCG), i.e., the probability of finding an entity under investigation before position *k* in the ranking as a function of *k*. Panel **(B)** shows the receiver operating characteristic (ROC) curves for the Random Forest model for each network type. Note that in both figures, the results of the complete network and the subjective-only network overlap completely

Then, we systematically assess the extent to which different centrality rankings provide useful information about the entities under investigation. We do it by measuring the normalized discounted cumulative gain (NDCG) [47], as described in Sect. 4.3. We find that degree centrality, measured in both the subjective and complete networks, is the most effective in finding entities involved in investigations (i.e., it has the highest NDCG) compared to the other centrality measures (Fig. 7A). NDCG can be interpreted as the probability of finding an entity under investigation before position *k* in the ranking, and this probability is above 20% for degree centrality in the top 100 node ranking, while it remains around 10% for the other centrality measures. As expected, since entities involved in objective transactions generally play peripheral roles, the NDCG for the network containing objective transactions was lower, ranging from 0 to 0.03%.

Lastly, we analyze which network type is most suitable for predicting whether an entity is under investigation. To this end, we use a logistic regression model and a Random Forest classifier, as described in Sect. 4.3. Before evaluating predictive performance, we assessed the correlation structure among the three predictors using Spearman correlation [49]. In the Complete and Subjective networks, degree and betweenness centrality are moderately correlated (ρ=0.67 and 0.66, respectively), while their correlation is stronger in the Objective network (ρ=0.76). Eigenvector centrality is generally independent of the other two measures across all networks ($\rho \leq 0.29$), indicating that multicollinearity is not severe.

The logistic regression yields out-of-fold AUC scores of 0.732, 0.736, and 0.591 for the Complete, Subjective, and Objective networks, respectively. The Random Forest achieves AUC scores of 0.755, 0.748, and 0.596. For the Objective network, the two models perform comparably (DeLong test: Z=−0.354, p=0.723). For the Complete and Subjective networks, the Random Forest outperforms logistic regression (Complete: Z=−6.955, p<0.001; Subjective: Z=−3.539, p<0.001), suggesting that centrality measures carry a non-linear predictive structure that a linear model cannot fully capture. Accordingly, we base the following network comparisons on the Random Forest predictions.

To formally compare networks, we applied the DeLong test on the Random Forest out-of-fold predictions. The Complete network slightly but significantly outperforms the Subjective network (Z=−3.593, p<0.001), while both significantly outperform the Objective network (Complete vs. Objective: Z=−8.697, p<0.001; Subjective vs. Objective: Z=−5.738, p<0.001). Figure 7B shows the ROC curves for each network type. Overall, the complete and subjective networks offer better predictive performance than the objective network. However, the overall predictive power is moderate, suggesting room for further improvement with a richer feature space. Although the difference between the Complete and Subjective networks is statistically significant, it is negligible in absolute terms (ΔAUC = 0.007) and unlikely to be of practical relevance for investigative prioritization. We therefore conclude that objective STRs contribute structurally (in terms of connectivity) but add no meaningful informational signal for entity-level detection.

## Conclusion

As criminal organizations continue to evolve their methods for concealing illicit financial transactions, increasingly sophisticated anti-money laundering (AML) frameworks are becoming necessary. To adhere to updated regulations, reporting entities are producing ever more unusual transaction reports, generating rich yet noisy data. Assessing the effectiveness of AML regulations is therefore essential to balance two competing risks: overwhelming investigators with excessive reporting, or overlooking signals that may be relevant for uncovering financial crime. This study evaluated the investigative value of objective suspicious transaction reports (STRs), which are often automatically generated based on pre-defined criteria. We defined the investigative value of objective STRs as their measurable contributions to: (i) *entity connectivity*, i.e., increasing the number of unique entities linked to investigated cases, (ii) *dossier connectivity*, i.e., joining previously disconnected dossiers into the main investigative network, and (iii) improving the *predictive accuracy* for identifying entities under investigation.

Our results show that, despite representing only 6.4% of all STRs, objective reports expand the investigative landscape. Incorporating them into the entity network added 15,403 unique entities (+9.3%), including 83 entities already under investigation, and increased the size of the giant component by 3529 nodes (+7.2%). At the dossier level, objective STRs connected 6816 additional dossiers (+13%), 235 of which contained investigated entities, and enlarged the dossier giant component by 1479 dossiers (+9.7%).

In contrast, predictive modeling based on centrality measures shows that objective STRs alone have limited capacity to identify investigated entities (ROC AUC = 0.60), compared to complete and subjective-only networks (both ROC AUC = 0.75). Although the difference between the Complete and Subjective networks is statistically significant (p<0.001), the absolute gain is negligibly small (ΔAUC = 0.007) and of no practical relevance for investigative prioritization.

Taken together, these findings suggest that the primary contribution of objective STRs lies in extending investigative reach and connecting dossiers, rather than enhancing direct detection of key actors. Rather than being discarded, their utility could be improved by enriching them with contextual information or by prioritizing them in typologies where threshold-based detection aligns with known laundering practices.

From a criminological perspective, these results highlight a central tension: the balance between the breadth of surveillance versus investigative depth. Automated reports ensure coverage, but without context, they risk adding noise; subjective reports, on the other hand, reflect human judgment, but may miss peripheral actors. The system-wide reporting burden, carried by institutions, the FIU, and law enforcement, must therefore be weighed against the modest investigative gains of objective reports. With the forthcoming EU AML package [53], which harmonizes suspicious reporting across member states and expands reporting obligations, these trade-offs will become even more important to monitor.

In response to our research question, we conclude that objective STRs broaden the scope of investigations and provide structural value by strengthening the connective fabric of suspicious transaction networks. Yet their added value for combating money laundering remains limited in their current form. Their role, therefore, is less about replacing subjective reporting than about complementing it, ensuring that automated detection contributes to, but does not overwhelm, the criminological fight against financial crime.

### Limitations

This work has several limitations that provide important avenues for future research and underline the need for continued evaluation of suspicious transaction reporting systems as AML regulations evolve.

First, the analysis is restricted to reported suspicious transactions. We do not observe non-reported (i.e., non-suspicious) transactions, which limits our ability to compare suspicious networks against a baseline of routine financial activity. Without such a baseline, we cannot assess relative anomaly levels, nor can we evaluate false positives or false negatives. Moreover, suspicious transaction reports are often generated in the context of ongoing intelligence work or emerging investigations. As a result, the observed network structures partly reflect investigative focus and reporting practices, rather than underlying criminal behavior alone. This limits the extent to which structural features can be directly attributed to money laundering activity. Therefore, the structural knowledge gained through network science must be combined with contextual knowledge [54].

Second, the empirical scope is limited to FIOD investigations. Our analysis focuses exclusively on investigations conducted by the Dutch Investigation Service for Financial and Tax Crime (FIOD), which represents only one part of the broader enforcement landscape. Other actors, including the Dutch police and additional national law enforcement agencies, also make extensive use of STRs and may apply different investigative strategies, priorities, and case-linking practices. Consequently, the relative investigative value of objective and subjective STRs may differ across institutional contexts. Our findings should therefore be interpreted as specific to the FIOD setting.

Third, increased network coverage does not necessarily imply increased investigative usefulness. While the inclusion of objective STRs expands network coverage and connects additional entities and dossiers, this does not automatically translate into practical investigative value. An increased volume of information with limited contextual detail may impose additional analytical burdens on investigators and reduce usability in practice. This suggests a need for improved reporting guidelines or richer contextual information, rather than rejecting objective reporting. At the same time, limited direct use in investigations does not preclude indirect effects, such as deterrence or broader situational awareness.

Fourth, the predictive analysis relies on relatively simple network measures. We focus on standard centrality measures, which were selected for their interpretability in the AML context. Degree centrality reflects transaction volume, betweenness identifies potential intermediaries or facilitators, and eigenvector captures influence through association with well-connected entities. Our results do not rule out the potential value of objective STRs when combined with other information. It is worth noting that the logistic regression model is not intended to provide an automated tool for flagging individuals for investigation. Instead, it serves as a methodological instrument to assess whether objective STRs contribute additional informative value for investigative decision-making at the aggregate level; its output should therefore be interpreted as evidence about the relative value of different reporting regimes, rather than as a basis for operational decisions on individual entities.

Looking ahead, the dataset contains rich metadata that could be leveraged alongside network centrality measures to improve predictive performance, opening new opportunities for investigating different types of actors in the money laundering practice. Among the most promising features are transaction amounts, which could be operationalized as edge weights, thereby distinguishing high-value hubs from structurally central but low-volume nodes. The present study intentionally focuses on how the overall pattern of subjective and objective transactions shapes network connectivity, rather than attributing investigative meaning to individual transactions for individual nodes. Transaction frequency could similarly inform temporal centrality measures, capturing nodes that are persistently central across time windows rather than transiently so. Entity types (e.g., individuals vs. companies vs. reporting entities) are particularly relevant because different actor categories are known to occupy structurally distinct roles in money laundering networks. Future work could explore more advanced modeling approaches, such as graph neural networks [55], with improved performance at the cost of interpretability, which is a practical consideration in operational AML contexts.

## Data Availability

The data that support the findings of this study are available from Fiscal Information & Investigation Service (FIOD), but restrictions apply to the availability of these data, which were used under an agreement for the current study and so are not publicly available.

## Footnotes

1 The Dutch Penal Code articles 420bis/ter/quater/quinqies referring to money laundering can be found at the url https://wetten.overheid.nl/jci1.3:c:BWBR0001854&boek=Tweede&titeldeel=XXXA&z=2010-10-10&g=2010-10-10.

2 See point 15 in the published FATF 40 recommendations at https://www.fatf-gafi.org/content/dam/fatf-gafi/recommendations/FATF%2DRecommendations%2D1990.pdf.

3 https://www.fiu-nederland.nl/.

4 https://www.fiod.nl/.

5 The complete list of institutions legally obliged to report can be found on the FIU website https://www.fiu-nederland.nl/en/home/supervisors/.

## Code availability

The code associated with this research is publicly available at the following URL: https://github.com/elenacandellone/suspicious-transactions-subjective-objective. A persistent version is deposited in Zenodo at the following URL: https://doi.org/10.5281/zenodo.20813218. This repository includes the code implementations of the methods discussed in the paper.
